# Improved geometric accuracy of whole body diffusion-weighted imaging at 1.5T and 3T using reverse polarity gradients

**DOI:** 10.1038/s41598-022-15872-6

**Published:** 2022-07-08

**Authors:** T. Sjöholm, J. Kullberg, R. Strand, M. Engström, H. Ahlström, F. Malmberg

**Affiliations:** 1 Department of Surgical Sciences, Uppsala University, Uppsala, Sweden; 2 Antaros Medical AB, Mölndal, Sweden; 3 Department of Information Technology, Uppsala University, Uppsala, Sweden; 4 Applied Science Laboratory, GE Healthcare, Uppsala, Sweden

**Keywords:** Whole body imaging, Magnetic resonance imaging

## Abstract

Whole body diffusion-weighted imaging (WB-DWI) is increasingly used in oncological applications, but suffers from misalignments due to susceptibility-induced geometric distortion. As such, DWI and structural images acquired in the same scan session are not geometrically aligned, leading to difficulties in e.g. lesion detection and segmentation. In this work we assess the performance of the reverse polarity gradient (RPG) method for correction of WB-DWI geometric distortion. Multi-station DWI and structural magnetic resonance imaging (MRI) data of healthy controls were acquired at 1.5T (n = 20) and 3T (n = 20). DWI data was distortion corrected using the RPG method based on b = 0 s/mm^2^ (b0) and b = 50 s/mm^2^ (b50) DWI acquisitions. Mutual information  (MI) between low b-value DWI and structural data increased with distortion correction (*P* < 0.05), while improvements in region of interest (ROI) based similarity metrics, comparing the position of incidental findings on DWI and structural data, were location dependent. Small numerical differences between non-corrected and distortion corrected apparent diffusion coefficient (ADC) values were measured. Visually, the distortion correction improved spine alignment at station borders, but introduced registration-based artefacts mainly for the spleen and kidneys. Overall, the RPG distortion correction gave an improved geometric accuracy for WB-DWI data acquired at 1.5T and 3T. The b0- and b50-based distortion corrections had a very similar performance.

## Introduction

Whole-body diffusion-weighted imaging (WB-DWI) is increasingly being used in oncological applications and has proven a valuable diagnostic tool in cancers such as multiple myeloma, melanoma, lymphoma and metastatic disease, as well as in screening applications^1,2^. Usage areas span tumor detection, tumor characterization and therapy response assessment^3,4^. Widespread clinical use of WB-DWI has however been limited and the technique is primarily used in specialized centers. One reason is that DWI data sets are typically affected by distortions and misalignments, meaning DWI and structural images acquired in the same scan session are not geometrically aligned. This mismatch can lead to difficulties when segmenting and characterizing organs and lesions in DWI and apparent diffusion coefficient (ADC) data sets, manually and automatically^2,3^. This also hinders the integration of DWI data into workflows for large-scale analysis of multi-parametric oncology data, in which geometric alignment between input images are essential for automatic and correct tumor evaluation^5–7^.

DWI visualizes differences in water diffusivity by incorporating diffusion sensitizing gradients into the magnetic resonance (MR) sequence. The b-value, set by the strength and timings of these gradients, makes the sequence sensitive to different scales of microscopic motion. In WB-DWI, two or three b-values are in general collected and followed by mono-exponential calculation of quantitative ADC maps^1^. To obtain ADC maps less sensitive to perfusion effects, while keeping a reasonable acquisition time and sufficient signal-to-noise ratio (SNR), the lowest acquired b-value for WB-DWI tends to lie in the range 50–100 s/mm^2^ ^1,8^. Currently, the most widely used sequence for WB-DWI image acquisition is diffusion-prepared single-shot spin echo planar imaging (EPI), collected with short-TI inversion recovery (STIR) fat suppression and in free breathing^1^. Multiple stations are collected to cover the head to mid-thigh and stations are combined into single volumes as a post-processing step. EPI has the advantage of a fast readout and as such it is less sensitive to macroscopic patient motion. The sequence is however sensitive to static magnetic field inhomogeneity, resulting in image distortions due to magnetic field susceptibility differences between tissues and at tissue air transitions^9^. The distortion occurs because of the EPI sequence’s low bandwidth in the phase encoding direction, with the magnetic field inhomogeneity causing signal phase shifts during readout. Susceptibility-induced distortion is the same regardless of applied b-value^10^ and the effect is more pronounced at higher field strengths^11^. As the static magnetic field inhomogeneity is dependent on the subject within the field-of-view (FOV), it can also give central frequency shifts between acquired stations. A second, separate, source of distortion is eddy currents, modifying the applied gradient fields and resulting in an image distortion that is typically dependent on the applied b-value^10^. Being a multi-station acquisition, WB-DWI can also suffer from inter-station misalignments due to patient movement.

A number of post-processing methods for correction of susceptibility-induced geometric distortion have been proposed, most developed for brain imaging applications. These include B_0_ field mapping^12^, registration approaches^13^ and usage of a reverse polarity gradient (RPG) acquisition^14,15^. For body applications, less has been evaluated. The RPG method has however been tested in e.g. prostate^16,17^, cervix^18^, breast^19,20^ and head and neck^21^ applications with good outcomes, while field mapping has been used in prostate imaging^22^ with improved geometric accuracy as a result. The usage of the RPG method has been described as more robust to large distortions compared to field mapping^15^. For geometric improvements of WB-DWI, Blackledge et al.^23^ corrected inter-station misalignments by aligning stations using histogram matching of overlapping slices. Whole station images were translated in the phase-encode direction giving improved WB-DW images in terms of alignment of structures at station boundaries. Alignment to structural images was not assessed. Ceranka et al.^24^ assessed rigid and deformable registration approaches for aligning WB-DWI and structural data acquired in the same scan session. In addition to these approaches, it has been shown that volume shimming can potentially be omitted at 1.5T, with the center frequency set equal for all WB-DWI stations^25^. Due to larger susceptibility-induced distortions, however, this approach cannot be used at 3T^26^.

The aim of this work was to assess the performance of the RPG method for correction of susceptibility-induced geometric distortion of WB-DWI healthy volunteer data acquired at 1.5T and 3T. The performance was assessed in terms of geometrical alignment between DWI and structural data, impact on ADC values and visual evaluation of image quality. The RPG method was developed for b = 0 s/mm^2^ (b0) DWI data. As ADC maps less sensitive to perfusion are recommended for clinical use we have, for clinical applicability, also assessed whether b = 50 s/mm^2^ (b50) DWI data can be used for RPG correction instead of the standard b0 data.

## Methods

### Subjects

Twenty-six healthy volunteers were included in this prospective study, with 20 subjects scanned at 1.5T (age mean 46.8 ± 15.4 years, body mass index mean 26.4 ± 4.5 kg/m^2^, 8 females) and 20 subjects scanned at 3T (age mean 50.1 ± 16.9 years, body mass index mean 25.4 ± 4.0 kg/m^2^, 12 females). 14 subjects were dually scanned at 1.5T and 3T. Study approval was obtained from the regional ethics committee (Uppsala Regional Ethics Review Board, ethics approval 2017/524) and all participants gave their written informed consent to participate. The study was conducted in accordance to the relevant guidelines and regulations.

### Data acquisition

Images were acquired at 1.5T (Achieva, Philips Healthcare, Best, The Netherlands, gradient system: 33 mT/m maximum amplitude, 180 T/m/s maximum slew rate) and at 3T (Signa PET/MR, GE Healthcare, Milwaukee, WI, USA, gradient system: 44 mT/m maximum amplitude, 200 T/m/s maximum slew rate). To reduce gut mobility, subjects fasted for 4 h and an intramuscular injection of Buscopan (2 mg/mL, 1 mL injection) was administered prior to scanning. For both scanners, DWI and structural data were collected station-wise using head volume and phased array surface coils. Five or six axial stations were collected per subject to cover the head to mid-thigh. DWI data was acquired using a diffusion-weighted spin echo EPI sequence with STIR fat suppression in free breathing^27^, while an axial T_1_-weighted Dixon scan was acquired for anatomical reference (Table 1). For the standard EPI sequence, phase encoding was in the anterior–posterior (AP) direction. For RPG distortion correction, a reverse EPI sequence was acquired immediately following the standard sequence with phase encoding in the posterior-anterior (PA) direction.

**Table 1:** MR image acquisition parameters at 1.5T and 3T.

Sequence	1.5T			3T		
	DWI		T1w Dixon	DWI		T1w Dixon
Sequence details	EPI, AP	EPI, PA	mDIXON	EPI, AP	EPI, PA	LAVA-Flex
Respiration	Free-breathing		Mixed^a^	Free-breathing		Mixed^a^
Slices per station (n)	40		96	38		100
Overlapping slices (n)	3		8	5		23
Fat suppression	STIR		–	STIR		–
Parallel imaging factor	2.5		2	2		2
TR (ms)	5600		5.5	3500		4.1
TE (ms)	73		1.7/3.7	61.7		1.7
TI (ms)	180		–	245.9		–
Flip angle	90		15	90		12
FOV (mm)	440 × 361		400 × 400	440 × 352		500 × 450
Acquired matrix	128 × 103		200 × 200	128 × 96		256 × 212
Slice thickness (mm)	6		5	6		5
Receiver bandwidth (Hz/pixel)	2719		587	1953		1302
b-values (s/mm^2^)	0, 50, 400, 900	0, 50	–	0, 50, 400, 900	0, 50, 900^b^	–
NSA	2, 2, 4, 9	2, 2	1	2, 2, 4, 9	2, 2, 4	1
Acquisition time	4:29 min	0:50 min	19 s	3:09 min	1:35 min	16 s

*SS-EPI* single shot echo planar imaging, *AP* anterior–posterior, *PA* posterior-anterior, *NSA* number of signal averages.

^a^Breath-hold for neck, chest and abdomen stations, free-breathing for head, pelvis and legs stations.

^b^b900 added to get an equal number of segments for the AP and PA acquisitions, enabling the same TR to be set.

ADC maps were calculated station-wise from b50, b = 400 s/mm^2^ (b400) and b900 images using a mono-exponential log-linear least square fit (MATLAB R2019b, the MathWorks, Inc., Natick, Massachusetts, US). DWI and ADC data were combined into single volumes in MATLAB. Overlapping slices were removed, with an equal number of slices removed from adjacent stations, and no intensity blending was performed.

### Geometric distortion correction

**Figure 1:**
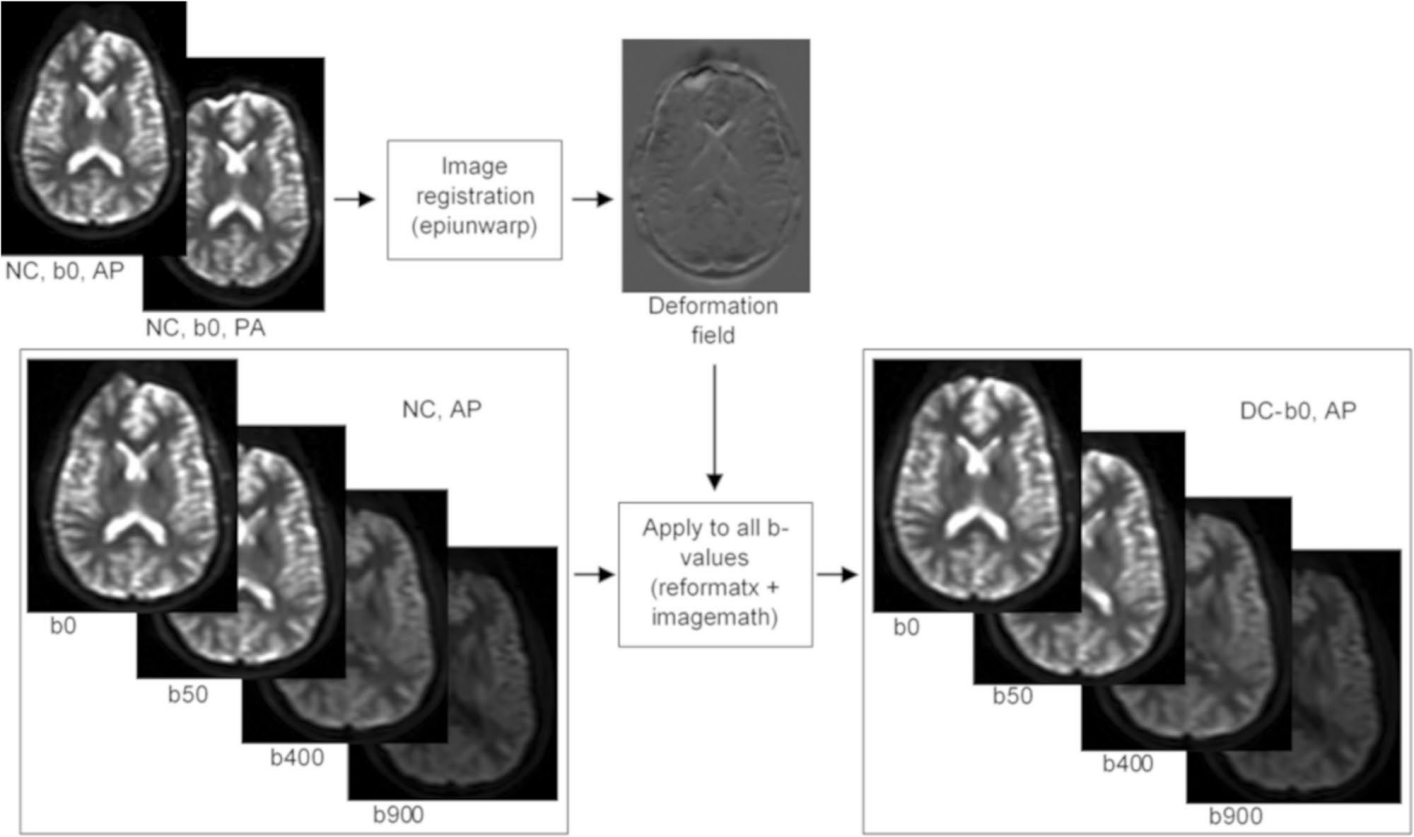
Illustration of the steps involved in the RPG geometric distortion correction. The CMTK tools epiunwarp, reformatx and imagemath were used as indicated. Epiunwarp calculates the 3D deformation field map from b0 AP and PA input images. The resulting deformation map then corrects all b-value images using the reformatx and imagemath tools. Reformatx applies the deformation map to each b-value image, while a voxel-wise multiplication with the Jacobian of the deformation is carried out using imagemath, correcting for signal pile-up.

The steps involved in the geometric distortion correction are illustrated in Fig. 1. The RPG method for correction of susceptibility-induced geometric distortion exploits the fact that two EPI data sets acquired with opposing phase-encoding gradients exhibit exactly opposite distortions. An undistorted data set can then be calculated by finding the mid-way between the two distorted data sets. In this work distorted DW images were corrected as described by Holland et al.^15^. In short, AP and PA b0 images are iteratively aligned using nonlinear registration in a multi-resolution framework in the form of decreasing Gaussian smoothing kernels. The obtained 3D deformation field map contains the voxel-wise displacement in the phase-encoding direction. As higher b-value data acquired in the same scan session exhibit the same susceptibility-induced geometric distortion as b0, the deformation map can be applied to the whole diffusion data set.

The Computational Morphometry Toolkit (CMTK) open source software^28^ was used for station-wise geometric distortion correction. The tools epiunwarp, reformatx and imagemath were combined in MATLAB for batch processing (Fig. 1). Regularization parameters were set to 0 (λ_1_) and 1000 (λ_2_), and the Gaussian smoothing kernel was set to start at a width of 16 mm, decreasing in steps of 0.25 mm until achieving original image resolution. At each smoothing level the number of optimization iterations was set to 5. The 1.5T images were rescaled to have approximately the same intensity range as the 3T images, meaning the same regularization parameters could be used for both scanners. The resulting deformation map was used to correct b0, b50, b400 and b900 images. To assess the feasibility of using b50 data instead of b0 data, the distortion correction was repeated with AP and PA b50 images and the resulting deformation map used to correct b50, b400 and b900 images.

### Evaluations

#### Geometrical alignment

The geometric correspondence between b0 and structural data was assessed using the mutual information (MI) similarity metric (MATLAB)^29^. Structural images were resampled using linear interpolation to match the resolution of the diffusion images. MI between b0 and resampled structural images was calculated station-wise for non-corrected and distortion corrected data. Each station was assigned to an anatomical region; head & neck, neck & chest, abdomen, abdomen & pelvis or pelvis & legs.

All image data was screened for incidental findings by a radiologist (20 y experience in WB-DWI). Benign abnormalities including cysts, gallstones and scoliosis were found at 1.5T (n = 6) and 3T (n = 7). These structures were manually segmented in 3DSlicer^30^, independently on structural MR, non-corrected DWI and distortion corrected DWI. For DWI segmentation, b50 (cysts and gallstones) or b900 (scoliosis) images were used depending on which data set gave the best image contrast for a given abnormality. For scoliosis, the spinal canal in the cervical and thoracic regions was segmented. Structural region of interest (ROI) data was resampled using linear interpolation to match the DWI ROI resolution. The segmentations were evaluated with Dice Similarity Coefficient (DSC), Euclidean distance of geometric centers (ED) and Average Hausdorff distance (AVD), comparing non-corrected and distortion corrected DWI segmentations with their structural counterpart (MATLAB).

#### ADC values

To assess what impact the distortion correction had on ADC, ADC values were measured by manual ROI segmentation of healthy tissues (3DSlicer). Tissues were selected as to cover the whole body: cerebral cortex, cerebellum, liver (segment VI), spleen, kidneys, psoas muscle, vertebral disk, pelvic bone (body of ilium), urinary bladder and femur. ROIs were outlined on five consecutive axial slices for all tissues except for the cerebellum and vertebral disks. For the cerebellum, three consecutive axial slices were used, while five vertebral disks were segmented with one axially drawn ROI per disk (L1-L5). ROIs were drawn to include as much as possible of the tissue while excluding tissue borders and major vessels. Segmentations were first performed with access to all non-corrected whole body images (DWI, ADC and structural MR). These segmentations were then overlaid on distortion corrected DWI and ADC images, and adjusted to exclude tissue borders and major vessels if needed. For each subject, the non-corrected and corrected mean ADC (ADC_mean_) were extracted for each tissue.

#### Visual evaluation

Visual evaluation was performed on whole body b50 images in two steps assessing (a) spine misalignment at station boundaries and (b) overall image quality. Prior to evaluation, images were corrected for inter-station signal intensity differences by using histogram matching of overlapping slices^23^. Sagittal whole body images were visually inspected in terms of spine misalignment at station boundaries (3DSlicer). For each station boundary, in a whole body data set, the subject was scored 0 or 1 if the spine was aligned or not aligned. A total score for each subject was calculated by summing the scores from each station boundary, i.e. a perfectly aligned spine at all station boundaries gave a total score of 0, while non-alignment at all station boundaries gave a total score of 3. This was performed blinded, and independently, for non-corrected and distortion corrected data. Overall image quality was independently assessed by three radiologists (2, 7 and 20 years of experience with WB-DWI). Scrollable movie clips of axial b50 images were prepared with non-corrected and distortion corrected images side by side. Each radiologist scored the distortion corrected data set as having equal or worse overall image quality compared to the corresponding non-corrected data set. If a distortion corrected data set was scored as worse, the amount of artefacts introduced was specified on a 3-point scale: (1) minor artifacts, most likely not affecting clinical use, (2) moderate artifacts, renders the image of borderline clinical use or (3) severe artifacts, image not clinically useful. The radiologist was also asked to specify the body parts and/or tissues affected and the type of artefacts introduced.

#### Distortion correction based on b50 data

To evaluate whether b50 data can be used for RPG distortion correction instead of b0 data, the same evaluations as for the b0-based correction was performed: station-wise calculation of MI between b50 and structural images, comparison between DWI and structural images by means of ROI-based metrics of segmented abnormalities, assessment of the impact on healthy tissue ADC values, and visual evaluation of spine alignment and image quality.

#### Distortion correction of high b-value images

High b-value whole-body images in the range of 800–1000 s/mm^2^ tend to be used clinically for visual analysis^1^. The effect of the RPG distortion correction on these images was therefore assessed by station-wise calculation of MI between b900 and structural images, and visual evaluation of spine alignment and image quality.

#### Distortion at different field strengths

The ROI-based analysis of segmented abnormalities and visual evaluation of spine misalignment enabled the extent of distortion at 1.5T and 3T to be compared. For this purpose, a subset analysis was performed for subjects scanned at both field strengths (n = 14).

### Statistical tests

Paired metrics were compared using paired, two-sided, Wilcoxon signed-rank tests (significance level 0.05). Bland–Altman plots were used to assess comparability between non-corrected and corrected ADC values for segmented tissues.

## Results

**Figure 2:**
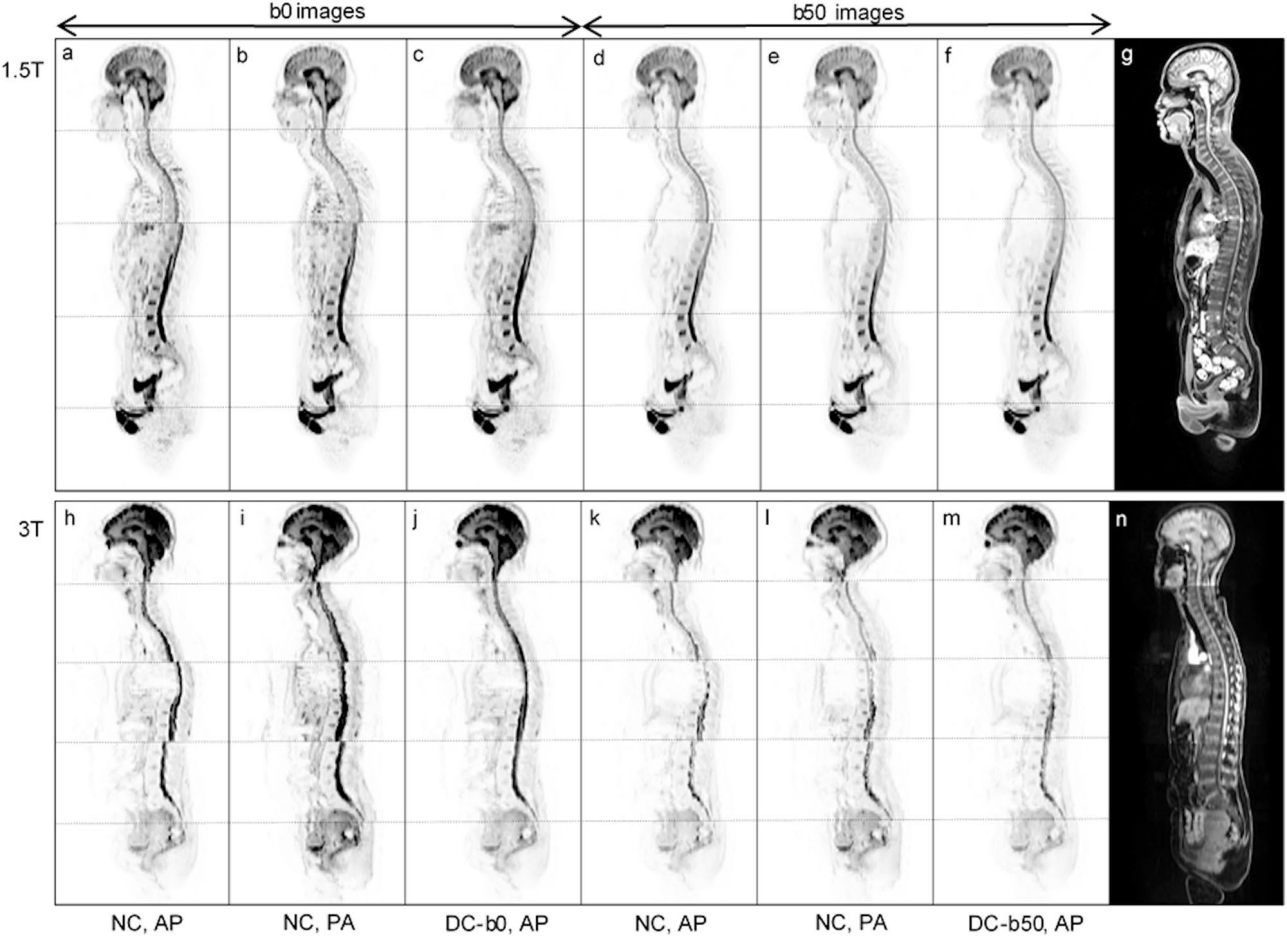
Representative examples of the susceptibility-induced distortion and the effect of distortion correction for sagittal b0 and b50 images at 1.5T (top row, **a**–**g**) and 3T (bottom row, **h**–**n**). For the b0 images, non-corrected (NC) anterior–posterior (AP) (**a**, **h**), NC posterior-anterior PA (**b**, **i**) and b0-based distortion corrected (DC-b0) AP (**c**, **j**) images are displayed. For the b50 images, NC AP (**d**, **k**), NC PA (**e**, **l**) and b50-based distortion corrected (DC-b50) AP (**f**, **m**) images are displayed. Corresponding structural images are also shown (**g**, **n**). DW images are shown in inverted grey-scale with station boundaries marked with a dotted line. Inter-station signal intensity correction was performed for the purpose of visualization^23^. Distortion is visible for the spine at station boundaries for NC images, and can be seen to be of opposing directions along the phase-encode direction for non-corrected AP and PA images. For the distortion corrected data the spine is linked at all station boundaries for both the b0- and b50-based corrections and its shape better matches that of the structural image.

Examples of the susceptibility-induced geometric distortion as observed for b0 and b50 sagittal WB-DW images at 1.5T and 3T are shown in Fig. 2. The distortion corrected images obtained using b0 and b50 as reference scans for the RPG distortion correction and corresponding structural images are also displayed. To visualize the effect of the RPG correction on higher b-value images, corresponding non-corrected and corrected b900 images are provided as Supplementary Fig. S1.

### Geometrical alignment

**Figure 3:**
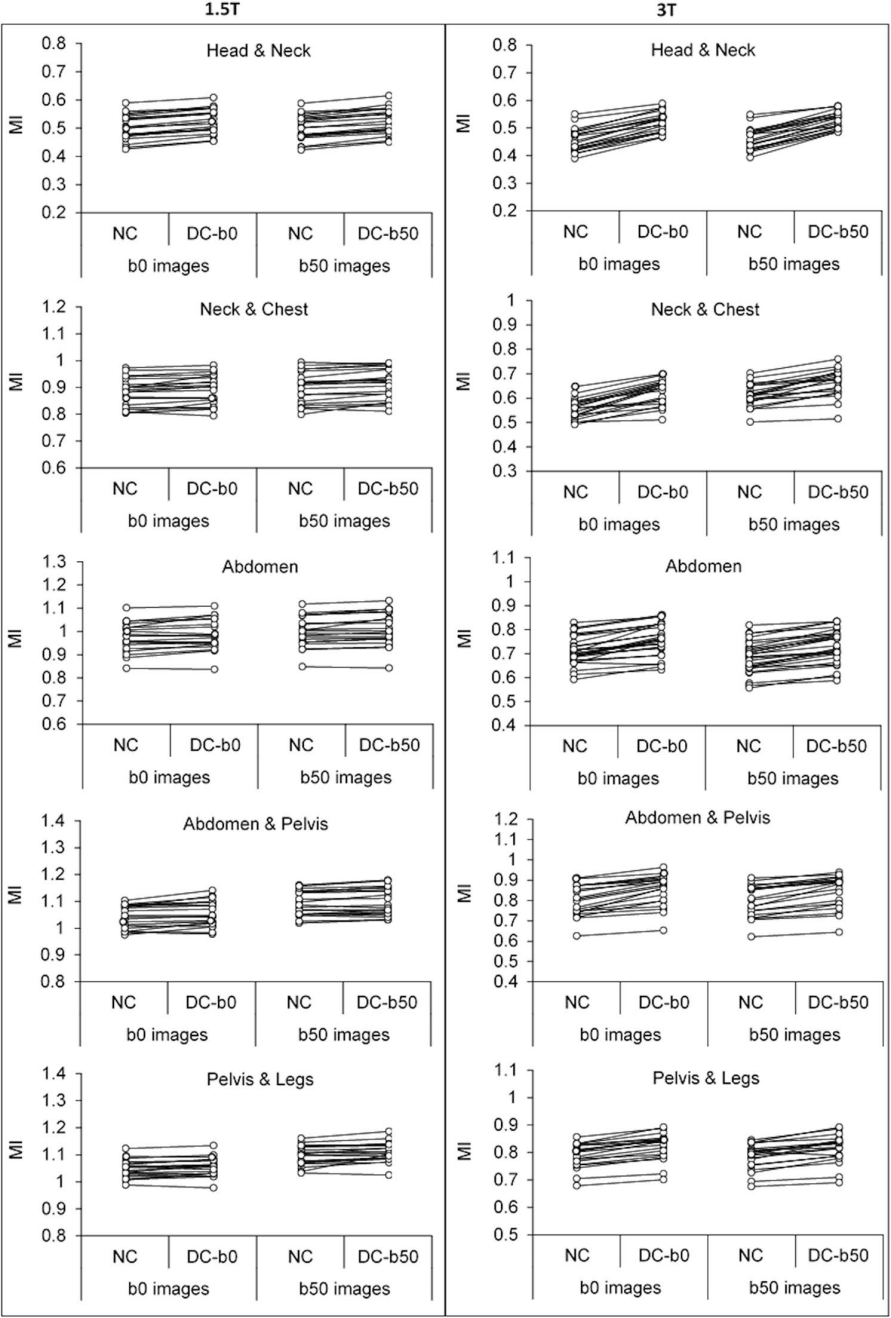
Mutual Information (MI) measured station-wise for non-corrected (NC), b0-based distortion corrected (DC-b0) and b50-based distortion corrected (DC-b50) data at 1.5T (left) and 3T (right). For the b0-based correction MI has been calculated between b0 and structural images, while for the b50-based correction MI has been calculated between b50 and structural images. An increased MI was obtained for the distortion corrected data for all anatomical regions (Wilcoxon signed-rank test, *P* < 0.05).

Overall, MI between b0 DWI and structural data increased when distortion correction was performed (Fig. 3). The distortion correction increased MI for all anatomical regions at 1.5T (head & neck: *P* < 0.0001, neck & chest: *P* = 0.0014, abdomen: *P* = 0.0057, abdomen & pelvis: *P* = 0.0014, pelvis & legs: *P* = 0.0060) and at 3T (all regions *P* < 0.0001).

When comparing DWI and structural data by means of ROI-based metrics, the largest improvements were obtained for spinal segmentations, while abdominal segmentations showed small or no improvement (Supplementary Table S1). Supplementary Fig. S2 shows examples of ROIs drawn at 1.5T and 3T for two subjects.

### ADC values

**Figure 4:**
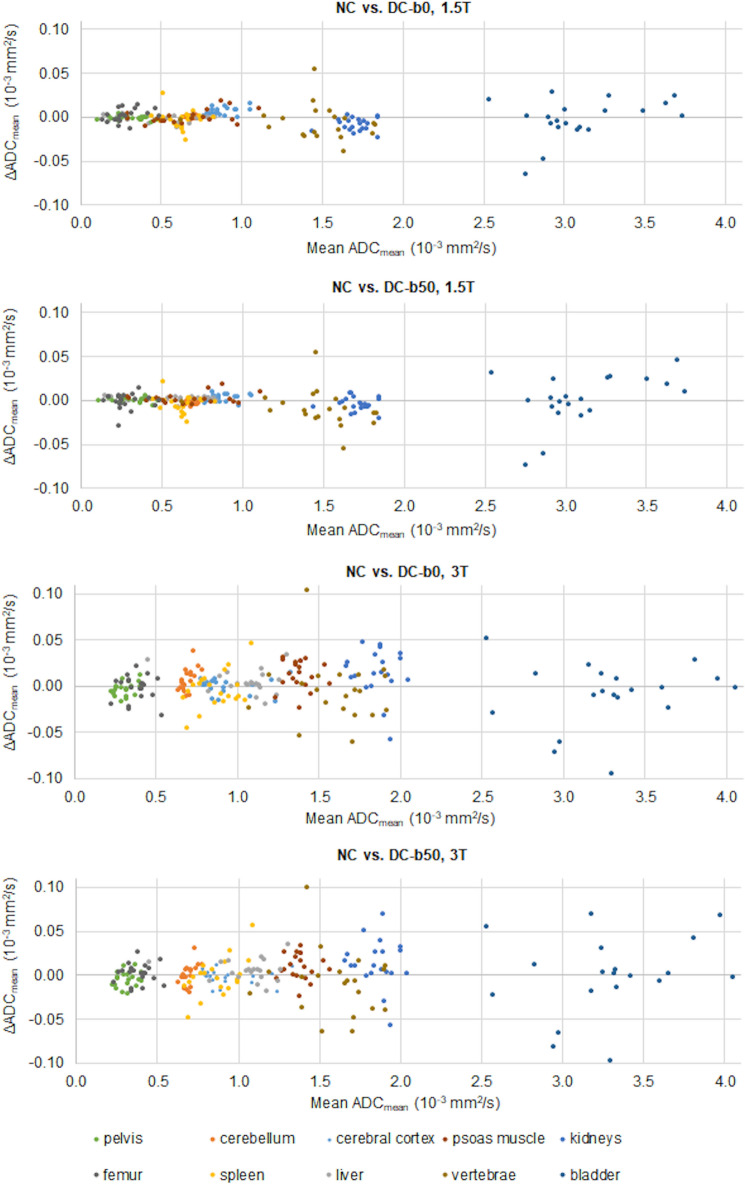
Bland Altman plots of the difference in ADC_mean_ (ΔADC_mean_) of healthy tissues measured between non-corrected (NC) and b0-based distortion corrected (DC-b0) data, and between NC and b50-based distortion corrected (DC-b50) data, at 1.5T and 3T. For the pelvis the body of ilium was segmented.

Bland–Altman plots of the difference in ADC_mean_ between non-corrected and b0-based distortion corrected measurements (ΔADC_mean_) are shown in Fig. 4. Overall, small numerical differences in ADC_mean_ were measured for all healthy tissues for both scanners, with ΔADC_mean_ values close to zero. The absolute median difference between non-corrected and corrected ADC_mean_ was small across all healthy tissues, but ADC bias assessed using paired Wilcoxon signed-rank tests identified a non-zero bias for the cerebral cortex (*P* = 0.0010) and kidneys (*P* = 0.00085) at 1.5T, and for the kidneys (*P* = 0.013), psoas muscle (*P* = 0.0077) and pelvis (*P* = 0.046) at 3T (Table 2). The number of adjusted ROIs for the distortion corrected data differed between scanners: at 1.5T the majority of ROIs was not adjusted (n = 14), while at 3T all subjects required at least one ROI update (Table 2).

**Table 2:** Median absolute ΔADC_mean_ measured for selected healthy tissues at 1.5T (top) and 3T (bottom) with the IQR in parentheses.

1.5T					
Tissue	NC vs DC-b0		NC vs DC-b50		ROI adjustments
	ΔADC_mean_ (10^–3^ mm^2^/s)	*P*	ΔADC_mean_ (10^–3^ mm^2^/s)	*P*	n
Cerebral cortex	0.0050 (0.0058)	**0.0010**	0.0035 (0.0040)	**0.0078**	0
Cerebellum	0.00078 (0.00074)	1.00	0.0028 (0.0018)	**0.031**	0
Liver	0.0014 (0.0040)	0.063	0.0028 (0.0016)	0.13	0
Spleen	0.0039 (0.0079)	0.19	0.0053 (0.0092)	**0.039**	6
Kidneys	0.0071 (0.010)	**0.00085**	0.0068 (0.0048)	0.10	0
Psoas muscle	0.0040 (0.0062)	0.44	0.0022 (0.0032)	0.31	0
Vertebral disk	0.013 (0.015)	0.15	0.013 (0.012)	**0.032**	2
Pelvis (body of ilium)	0.0019 (0.0021)	0.63	0.0021 (0.0025)	0.63	0
Urinary bladder	0.011 (0.014)	0.71	0.016 (0.021)	0.47	0
Femur	0.0041 (0.0079)	0.30	0.0044 (0.0048)	1.00	0

3T					
Tissue	NC vs DC-b0		NC vs DC-b50		ROI adjustments
	ΔADC_mean_ (10^–3^ mm^2^/s)	*P*	ΔADC_mean_ (10^–3^ mm^2^/s)	*P*	n
Cerebral cortex	0.0048 (0.0060)	0.79	0.0039 (0.0070)	0.56	0
Cerebellum	0.010 (0.010)	0.057	0.0073 (0.011)	0.75	1
Liver	0.0061 (0.011)	0.34	0.0064 (0.010)	0.086	3
Spleen	0.012 (0.011)	0.46	0.012 (0.013)	1.00	16
Kidneys	0.023 (0.023)	**0.013**	0.020 (0.025)	**0.027**	18
Psoas muscle	0.017 (0.017)	**0.0077**	0.012 (0.019)	**0.0087**	1
Vertebral disk	0.015 (0.018)	0.13	0.018 (0.032)	0.12	15
Pelvis (body of ilium)	0.0050 (0.0080)	**0.046**	0.0063 (0.0080)	**0.011**	3
Urinary bladder	0.013 (0.021)	0.30	0.016 (0.055)	0.84	8
Femur	0.0074 (0.012)	0.40	0.0074 (0.011)	1.00	3

The reported *P*-values were obtained from paired Wilcoxon signed-rank tests when comparing ADC_mean_ values extracted from NC and DC-b0 data, and from NC and DC-b50 data. Statistical significance is marked in bold (*P* < 0.05). The number of ROIs that had to be adjusted for the distortion corrected data is also shown: a total of 6 subjects required ROI adjustments at 1.5T, while all subjects required ROI adjustments at 3T.

*NC* non-corrected, *DC-b0* b0-based distortion correction, *DC-b50* b50-based distortion correction, *ADC*_mean_ mean apparent diffusion coefficient, *ROI* region of interest.

### Visual evaluation

**Figure 5:**
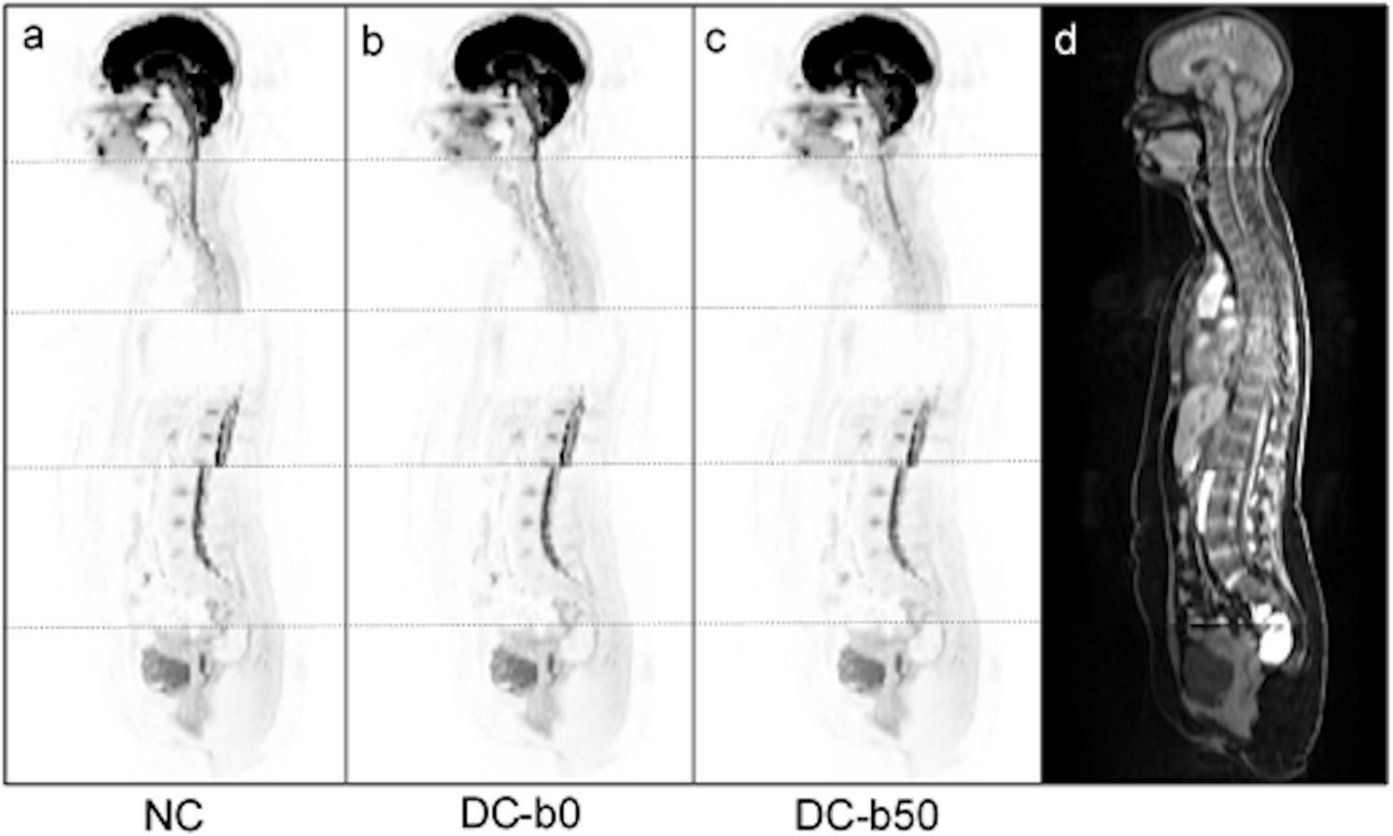
Example of a subject at 3T for which susceptibility changes cause both a signal void at the chest and large geometric distortion at the abdominal station boundary. Sagittal b50 anterior–posterior (AP) images are shown in inverted grey-scale: (**a**) non-corrected (NC), (**b**) b0-based distortion corrected (DC-b0) and (**c**) b50-based distortion corrected (DC-b50). The corresponding structural image is also shown (**d**). Station boundaries are marked with a dotted line and inter-station signal intensity correction was performed for the purpose of visualization^23^. The signal drop-out resulted in spine misalignment not being assessed at the chest station boundary. The geometric distortion seen as a spine misalignment at the abdominal station boundary could not be corrected by either the b0- or b50-based distortion corrections.

Distortion correction improved spine alignment at station boundaries at both field strengths (Table 3). At 3T, large signal dropout was seen for one subject at the neck and for two subjects at the chest (Fig. 5), meaning the total number of evaluable station boundaries at these locations were 19 and 18, respectively. Without distortion correction, 14 subjects (70%) had at least one station boundary with spine misalignment at 1.5T, with the majority of cases being in the neck (n = 13, 62%). After distortion correction, the spine was aligned at all station boundaries for all subjects. At 3T, all subjects had at least one spine misalignment for non-corrected data, with most discrepancies arising in the chest (n = 17, 94%). The distortion correction decreased the number of misalignments, but 5 subjects (25%) had one remaining misalignment after correction (Fig. 5). With distortion correction, the average misalignment score decreased from 1.1 to 0 at 1.5T, and from 1.8 to 0.3 at 3T.

**Table 3:** Visual assessment of spine misalignment at station boundaries for NC, DC-b0 and DC-b50 data, at 1.5T (top) and 3T (bottom). At both field strengths, b50 and b900 images are evaluated. The total number of subjects affected by spine misalignments are displayed, as well as the number of subjects with spine misalignments at the different anatomical locations along the spine (neck, chest and abdomen). In parentheses the total number of evaluable subjects are displayed. At 3T, large signal dropout for 1 subject at the neck and 2 subjects at the chest meant that the total number of station boundaries that could be evaluated at these locations were 19 and 18, respectively. The average misalignment score across all subjects is also given.

1.5T						
	b50			b900		
	NC	DC-b0	DC-b50	NC	DC-b0	DC-b50
Subjects affected by misalignment, *n*	14 (20)	0 (20)	0 (20)	15 (20)	1 (20)	2 (20)
Neck, *n*	13 (20)	0 (20)	0 (20)	14 (20)	0 (20)	0 (20)
Chest, *n*	8 (20)	0 (20)	0 (20)	8 (20)	1 (20)	2 (20)
Abdomen, *n*	0 (20)	0 (20)	0 (20)	3 (20)	0 (20)	0 (20)
Average misalignment score	1.1	0	0	1.3	0.1	0.1

3T						
	b50			b900		
	NC	DC-b0	DC-b50	NC	DC-b0	DC-b50
Subjects affected by misalignment, *n*	20 (20)	5 (20)	4 (20)	19 (20)	4 (20)	5 (20)
Neck, *n*	11 (19)	1 (19)	0 (19)	15 (19)	2 (19)	2 (19)
Chest, *n*	17 (18)	3 (18)	3 (18)	16 (18)	3 (18)	4 (18)
Abdomen, *n*	8 (20)	2 (20)	1 (20)	7 (20)	1 (20)	1 (20)
Average misalignment score	1.8	0.3	0.2	1.9	0.3	0.4

*NC* non-corrected, *DC-b0*  b0-based distortion correction, *DC-b50 *b50-based distortion correction.

**Figure 6:**
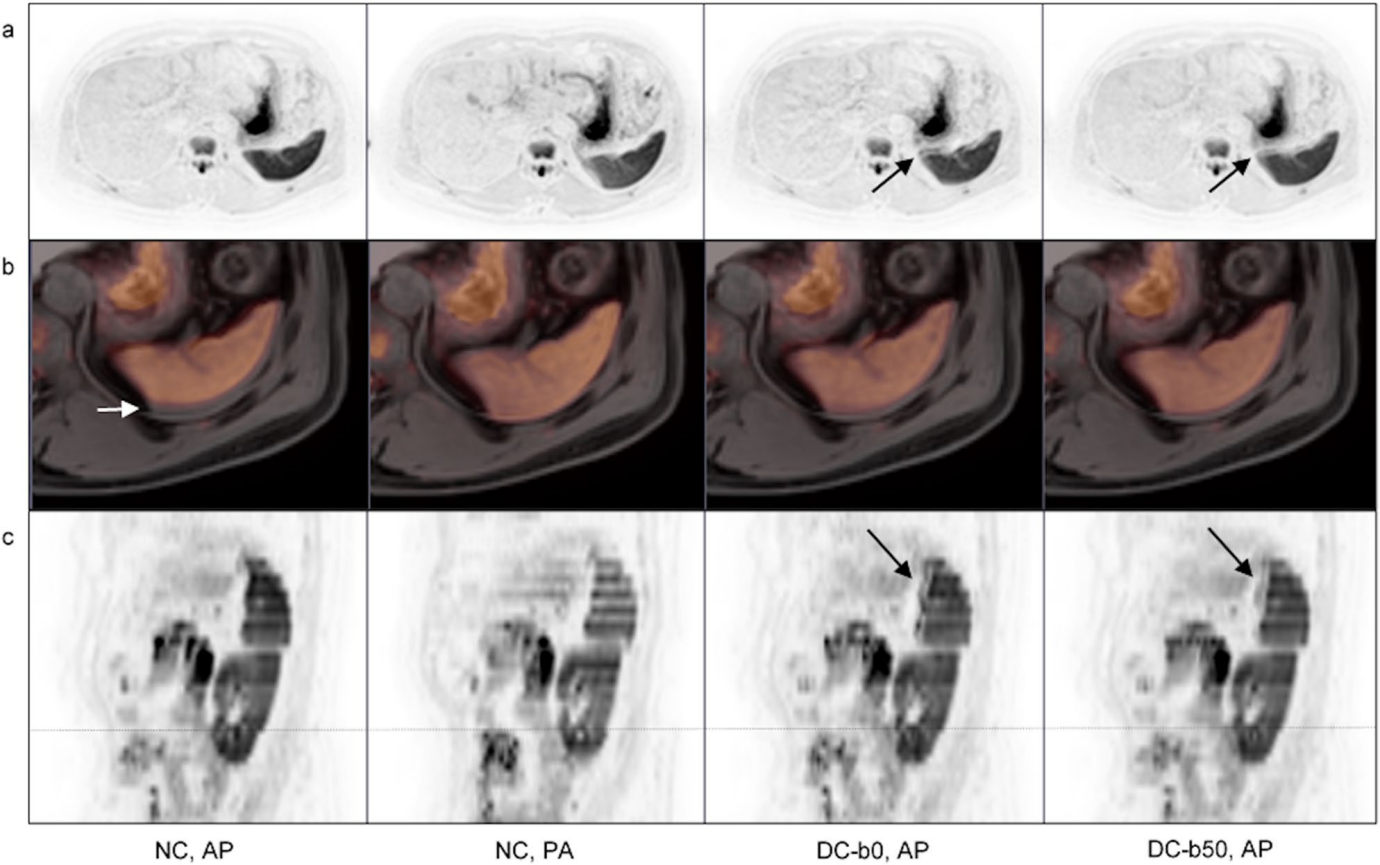
Typical registration artefacts caused by the RPG method as observed at 1.5T. Axial b50 images (**a**) axial b50 images overlaid on corresponding structural images (**b**) and sagittal b50 images (**c**) are shown. In each column (left to right) the non-corrected (NC) anterior–posterior (AP), NC posterior-anterior (PA), b0-based distortion corrected (DC-b0) AP and b50-based distortion corrected (DC-b50) AP images are displayed. Axial and sagittal b50 images in inverted grey scale show spleen registration artefacts as marked by arrows (**a**, **c**). Although artefacts are introduced, there is an improvement in the geometrical alignment. This is exemplified by zoomed axial b50 images in yellow color scale overlaid on corresponding structural images (**b**), with misalignment in the NC AP image indicated with an arrow. For the sagittal images the station border is marked with a dashed line. The radiologists’ assessments of overall image quality gave a mean score of 1.7 and 1.0 for the DC-b0 and DC-b50 images, respectively.

When assessing axial slices, the radiologists scored the overall image quality as worse for the majority of subjects at 1.5T (mean n = 15 subjects, 73%) and at 3T (mean n = 13 subjects, 63%) (Table 4). Worse image quality was attributed to minor artefacts in the majority of these subjects (score 1: 73% at 1.5T, 76% at 3T). Localized moderate or severe artefacts were however also present (score 2: 27% at 1.5T, 21% at 3T, score 3: 0% at 1.5T, 3% at 3T). Artefacts were mainly noted in the spleen and/or kidneys and the causes attributed to registration artefacts. Typical artefacts are shown in Fig. 6 (1.5T) and 7 (3T).

**Table 4:** Rating and scores given by three radiologists when comparing the image quality of non-corrected and distortion corrected axial b50 images side by side. The number of distortion corrected scans scored as ‘equal’ or ‘worse’ compared to the corresponding non-corrected scans are shown for DC-b0 and DC-b50 data. The scores given for scans marked as having worse image quality are also shown. The mean rating and score for the three readers’ assessments are indicated. In parentheses, percentages are given.

1.5T										
	NC vs DC-b0					NC vs DC-b50				
	Equal	Worse	Score 1	Score 2	Score 3	Equal	Worse	Score 1	Score 2	Score 3
Reader 1, n (%)	2 (10)	18 (90)	18 (100)	0 (0)	0 (0)	2 (10)	18 (90)	18 (100)	0 (0)	0 (0)
Reader 2, n (%)	5 (25)	15 (75)	10 (67)	5 (33)	0 (0)	2 (10)	18 (90)	14 (78)	4 (22)	0 (0)
Reader 3, n (%)	9 (45)	11 (55)	4 (36)	7 (64)	0 (0)	8 (40)	12 (60)	6 (50)	3 (25)	3 (25)
Mean, n (%)	5.3 (27)	14.7 (73)	10.7 (73)	4.0 (27)	0 (0)	4.0 (20)	16.0 (80)	12.7 (79)	2.3 (15)	1.0 (6)

3T										
	NC vs DC-b0					NC vs DC-b50				
	Equal	Worse	Score 1	Score 2	Score 3	Equal	Worse	Score 1	Score 2	Score 3
Reader 1, n (%)	5 (25)	15 (75)	15 (100)	0 (0)	0 (0)	3 (15)	17 (86)	17 (100)	0 (0)	0 (0)
Reader 2, n (%)	8 (40)	12 (60)	11 (92)	1 (8)	0 (0)	6 (30)	14 (70)	11 (79)	3 (21)	0 (0)
Reader 3, n (%)	9 (45)	11 (55)	3 (27)	7 (64)	1 (9)	6 (30)	14 (70)	7 (50)	5 (36)	2 (14)
Mean, n (%)	7.3 (37)	12.7 (63)	9.7 (76)	2.7 (21)	0 (3)	5.0 (25)	15.0 (75)	11.7 (78)	2.7 (18)	0.7 (4)

*NC* non-corrected, *DC-b0* b0-based distortion correction, *DC-b50* b50-based distortion correction.

### Distortion correction based on b50 data

The evaluations (geometric alignment, ADC values and visual assessments) of the b50-based geometric distortion correction were in line with that of the b0-based distortion correction. When b50 data was used for distortion correction, an increased MI was obtained for all body parts at 1.5T (head & neck: *P* < 0.0001, neck & chest: *P* = 0.00089, abdomen: *P* = 0.00062, abdomen & pelvis: *P* = 0.0057, pelvis & legs: *P* = 0.00072) and at 3T (head & neck, neck & chest, abdomen, and abdomen & pelvis: *P* < 0.0001, pelvis & legs: *P* = 0.00014) (Fig. 3). When comparing ROI-based metrics of segmented incidental findings on DWI with structural MR, larger improvements were seen for spinal segmentations compared to abdominal segmentations (Supplementary Table S1).

**Figure 7:**
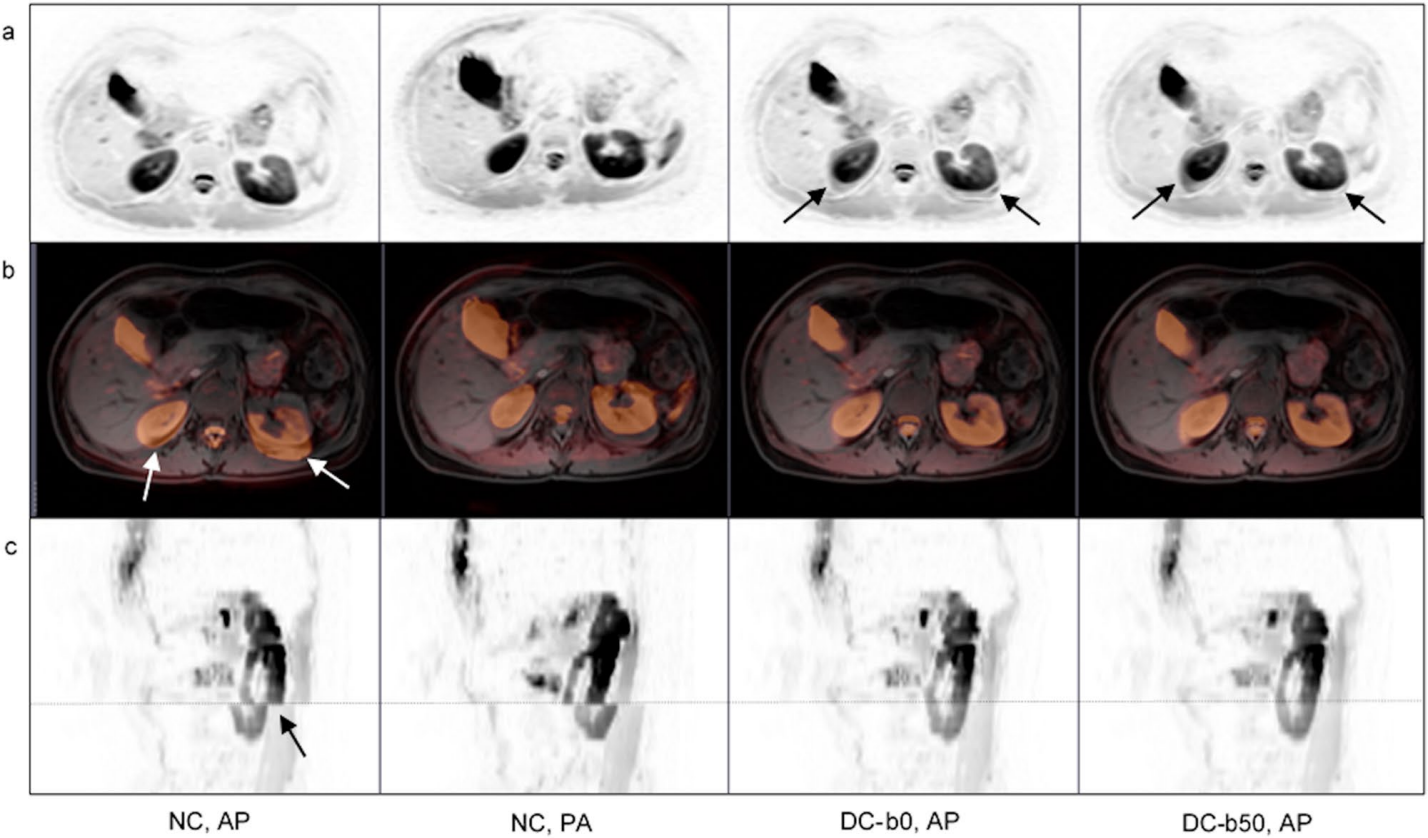
Typical registration artefact caused by the RPG method as observed at 3T. Axial b50 images (**a**) axial b50 images overlaid on corresponding structural images (**b**) and sagittal b50 images (**c**) are shown. In each column (left to right) the non-corrected (NC) anterior–posterior (AP), NC posterior-anterior (PA), b0-based distortion corrected (DC-b0) AP and b50-based distortion corrected (DC-b50) AP images are displayed. Axial b50 images in inverted grey scale show the kidney registration artefact as marked by arrows (**a**). Although artefacts are introduced, there is an improvement in the geometrical alignment. This is exemplified by zoomed axial b50 images in yellow color scale overlaid on corresponding structural images (**b**) and by sagittal b50 images in inverted grey scale (**c**). Misalignments in NC AP images are indicated with arrows in (**b**) and (**c**). For the sagittal images the station border is marked with a dashed line. The radiologists’ assessments of overall image quality gave a mean score of 1.0 and 1.7 for the DC-b0 and DC-b50 images, respectively.

Small numerical differences between non-corrected and distortion corrected ΔADC_mean_ measurements were seen (Fig. 4). ROI placement was the same as for the b0-based correction at both field strengths. After distortion correction, the spine was aligned for all subjects at all station boundaries at 1.5T, while 4 subjects (20%) had one remaining misalignment after correction at 3T (Table 3, Fig. 5). The radiologists scored the overall image quality as worse for the majority of subjects at 1.5T (mean n = 16, 80%) and at 3T (mean n = 15, 75%) (Table 4). As for the b0-based distortion correction, worse image quality was mainly attributed to minor artefacts (score 1: 79% at 1.5T, 78% at 3T), but localized moderate or severe artefacts were present (score 2: 15% at 1.5T, 18% at 3T, score 3: 6% at 1.5T, 4% at 3T). At both field strengths, artefacts were mainly noted in the spleen and kidneys, and were attributed to registration artefacts in all cases. Typical artefacts were similar for the b0- and b50-based distortion corrections (Figs. 6 and 7).

### Distortion correction of high b-value images

The RPG distortion correction gave small numerical changes in b900 station-wise MI at both field strengths (Supplementary Figure S3). The exception being the head & neck, for which a larger MI improvement was seen (*P* < 0.0001 for both field strengths and for both the b0- and b50-based distortion correction). At 1.5T, using the b0-based distortion correction, MI did not change for the pelvis & legs (*P* = 0.91), while a small systematic decrease in MI was measured for the neck & chest (*P* = 0.00013), abdomen (*P* = 0.00027) and abdomen & pelvis (*P* = 0.00029). Usage of the b50-based distortion correction gave better geometrical alignment between b900 and structural data at 1.5T: increased MI for the abdomen (*P* = 0.0034) and pelvis & legs (*P* = 0.036), and no change in MI for the neck & chest (*P* = 0.35) and abdomen & pelvis (*P* = 0.48). At 3T, the b0-based distortion correction gave an increased MI for the abdomen & pelvis (*P* = 0.0064) and pelvis & legs (*P* = 0.029), while no difference in MI was measured for the neck & chest (*P* = 0.18) and abdomen (*P* = 0.20). Again, the b50-based distortion correction gave a slight improvement in geometrical alignment to structural images: increased MI for the abdomen (*P* = 0.038), abdomen & pelvis (*P* = 0.00017) and pelvis & legs (*P* = 0.0026), and no change in MI for the neck & chest (*P* = 0.75). In general, the b900 images at 3T are of low SNR, which is evident in the larger spread of MI-values compared to the MI-value spread of b0 and b50 images (Fig. 3, Supplementary Fig. S3).

The visual assessments of b900 images gave results that were in line with the low b-value image evaluations. A small number of spine misalignments remained after b0- or b50-based distortion corrections (Table 3) and the radiologists mainly reported minor artefacts (Supplementary Table S2). At both field strengths, localized registration artefacts were noted and occurred mainly in the spleen. The artefacts had the same appearance as for the low b-value images (Figs. 6 and 7), with no new artefacts introduced.

### Distortion at different field strengths

Comparing DWI and structural data by means of ROI-based metrics, indicated larger distortions at 3T compared to 1.5T for non-corrected data; lower DSC, and larger ED and AVD were measured at 3T (Supplementary Table S1, Supplementary Fig. S2). The distortion correction was also seen to give larger improvements at 3T compared to 1.5T.

Visual assessment of spine misalignment at stations boundaries for subjects scanned at both field strengths (n = 14) are displayed per patient as Supplementary Table S3. A higher number of misalignments are seen at 3T, indicating larger distortions at this field strength.

## Discussion

In this work we assessed the performance of the RPG method for correction of susceptibility-induced geometric distortion of WB-DWI healthy volunteer data acquired at 1.5T and 3T. Geometric distortions were present in the majority of acquired data, with larger distortions observed at 3T compared to 1.5T. In terms of improved geometric accuracy, the RPG method performed well at both 1.5T and 3T: an increase in MI between low b-value DWI and structural MR data was measured for distortion corrected data, for all anatomical regions, and a lower number of spine misalignments was visually observed at station boundaries. Overall, improvements in geometrical accuracy could aid WB-DWI gain a wider clinical acceptance and is also important in other aspects; it would benefit the development of detection and segmentation techniques in particular for multispectral data and aid implementation for radiotherapy usage. An improved geometrical accuracy could also facilitate improved co-registration of WB-DWI data acquired at different time points, for example during treatment response assessments. Artefact-reduction has been described as a hurdle for usage of ADC as a primary endpoint biomarker^8^ and WB-DWI is not yet included in the Quantitative Imaging Biomarker Alliance (QIBA) DWI profile longitudinal claim statement^31^. Providing a reduction in image distortion could aid standardization efforts.

This study further showed that the RPG correction can be used with b50 EPI data as input, instead of the standard b0 EPI data. The susceptibility-induced distortion corrected by the RPG method is thought to be the same regardless of b-value^10^, giving validity to this approach. With regards to clinical usage, this is of importance as a perfusion insensitive lowest b-value is recommended and b0 data tend not to be collected. Although no comparison was made with a b0-based distortion correction, Winter et al.^21^ also showed that the RPG method using AP and PA b = 150 s/mm^2^ images reduces distortion in head and neck EPI-based DWI acquisitions.

The RPG distortion correction of high b-value images did not improve the overall geometrical alignment in terms of MI between b900 and structural images to the same extent as for b0 and b50 images. For stations covering the chest and abdomen, the correction gave a decreased or unchanged MI at 1.5T, and an improved or unchanged MI at 3T. Reasons for the poorer performance seen for b900 data are likely due to eddy current induced distortions and differences in motion between acquired b-value images. The b900 images are likely to be affected by eddy currents due to the strong gradient pulses used for data acquisition, and more likely to be affected by breathing motion artefacts due to the increased number of signal averages used in the acquisition (9 in the current study). The head & neck and pelvis & legs stations showed the largest improvements for b900 data, corresponding to stations less affected by breathing motion. In addition to these effects, high b-value images are inherently of low SNR, affecting the accuracy of the MI metric calculation. This latter effect was in particular seen at 3T, for which a larger MI-value spread was measured for b900 images compared to low b-value images. Although the overall geometrical alignment gave mixed results for b900 images, visual assessment of image quality showed that the RPG distortion correction did not produce additional artefacts for b900 images compared to low b-value images. Moreover, a localized improvement of the distortion correction was seen for the spinal cord at both field strengths, both in terms of a reduced number of spine misalignments and an improved geometrical alignment of incidental findings. This highlights that a potential clinical application of the distortion correction is likely to be application dependent.

Bland–Altman plots exhibited small numerical differences between non-corrected and corrected ADC_mean_ values for healthy tissues. Across all healthy tissues, a median absolute difference of 0.0040 × 10^–3^ mm^2^/s was measured for both the b0- and b50-based correction at 1.5T, while at 3T median absolute differences of 0.011 and 0.0095 × 10^–3^ mm^2^/s were measured for the b0- and b50-based corrections, respectively. Published results on test–retest ADC measurements in free-breathing WB-DWI are scarce, but Kwee et al.^32^ showed that the mean ADC bias of healthy liver parenchyma was in the range of − 0.01–0.09 × 10^–3^ mm^2^/s at 1.5T. The liver ADC bias measurements in this work can hence be considered small (mean absolute ΔADC_mean_ 0.003 × 10^–3^ mm^2^/s at 1.5T). An overall trend of larger ADC_mean_ differences was seen at 3T compared to 1.5T. To some extent, this is attributable to the ROI adjustment needed for distortion corrected data at 3T for which at least one ROI adjustment was needed per subject. The ADC bias assessment showed that the RPG method has the potential to introduce a non-zero bias, as observed for a subset of healthy tissues. This bias is however numerically very small in terms of ΔADC_mean_ and unlikely to have a clinical impact.

The visual assessment of spine misalignment at station boundaries showed that non-corrected datasets had a larger number of misalignments compared to corrected datasets. The shape of the whole spine improved after correction and better matched its structural counterpart, in particular at 3T. Visual assessment of axial b50 and b900 images showed that the RPG method can give rise to localized registration artefacts. This was particularly evident for the spleen and kidneys, corresponding to tissues exhibiting large distortions in this study. The spleen most likely as it lies close to the air-tissue interface of the lung, meaning large susceptibility-induced distortions in this area of the body. The kidneys were for many subjects imaged by two stations, meaning the kidney as a whole was affected by central-frequency shifts between stations. As a free-breathing WB-DWI acquisition was used, the abdominal organs are also affected by motion. For WB-DWI it has been shown that imaging in free-breathing is preferably used, giving an intra-voxel coherent motion pattern that exhibits an improved image SNR compared to breath-hold or navigator triggered imaging^33^. As the RPG method requires an additional image acquisition an inherent limitation of the technique is however the potential for gross subject movement, other than respiratory motion, between the AP and PA data acquisitions. To minimize the risk of this type of movement occurring, the PA acquisition followed immediately after the AP acquisition in the current study.

A limited number of incidental finding abnormalities were found and used to further assess the geometric correspondence between DWI and structural MR data. Overall, the distortion correction gave a numerical improvement in the ROI-based performance metrics assessed, in particular at 3T. The results however indicated that the improvement is position dependent: larger improvements were seen in the spine, but small or no improvement was seen for abdominal lesions. This is partly due to breathing motion differences between abdominal DWI and structural MR acquisitions, which was not corrected for in this study.

The extra scan time for the PA phase-encoded sequences in this study was relatively long as both b0 and b50 images were acquired: 0:50 and 1:35 min per station at 1.5T and 3T, respectively. In addition to an increased likelihood of gross subject motion, the additional scan time is a prohibitive factor to achieve patient compliance during the already long total scan time for whole body MR protocols. In practice, however, an EPI sequence giving only one set of images of PA phase-encoding is needed (e.g. b0 or b50), meaning the scan time can be reduced compared to our study protocol. For further time saving, it would also be possible to take advantage of the fact that the distortion corrected image can be composed from corrected AP and PA data sets. For the imaging protocol used in this study, this would entail acquiring low b-value images with an NSA of 1 for the AP and PA data acquisitions and as such the RPG method would give a negligible penalty in acquisition time compared to the standard protocol of acquiring one AP acquisition with an NSA of 2. Although not available at our imaging center, there are also sequences available that are optimized to allow for shorter acquisition times that have been implemented by some MR scanner manufacturers (e.g. PROGRES, GE Healthcare). The extra time needed for these purpose-built sequences is one TR (5.6 s at 1.5T and 3.5 s at 3T in the current study), making the RPG method more feasible for clinical applications.

A limited amount of work has been published on distortion correction of WB-DWI. Blackledge et al. however assessed a correction method based on histogram matching of overlapping slices^23^, while Ceranka et al. looked at image registration between DWI and structural images acquired in the same scan session^24^. Both approaches require the acquisition of overlapping slices and that inter-station intensity standardization is performed prior to correction. Intensity standardization can be problematic at 3T for which large signal dropout can be obtained and common approaches relying on signal intensity registration^23,34^ would not work adequately. An advantage of the RPG method is that no pre-processing of the acquired data is needed. In addition to these approaches, it has been shown that volume shimming can potentially be omitted at 1.5T, with the center frequency set equal for all WB-DWI stations^25^. During protocol development in this study this approach was however tested at 1.5T, but no improvements in station distortion was observed.

This study has some limitations. We did not take into account distortion due to eddy currents. Eddy currents have the potential to affect higher b-value images, in particular the b400 and b900 images acquired in this study. This can give rise to distortion in the images that are not present in lower b-value images, and can introduce errors in ADC measurements^9^. Clinically, high b-value images tend to be used for visual evaluation of WB-DWI^1^. Although the RPG method corrects for susceptibility-induced distortion in these images, the effect of eddy current distortion was not assessed in this work. Registration-based methods for eddy current correction have however been proposed^35,36^ and could be tested for WB-DWI. Modifications to these single-station brain registration algorithms are likely needed for utilization in multi-station WB-DWI. Furthermore, the RPG method is dependent on the scale of the distortion of input data. If the distortion is too large, the RPG method is not capable of restoring the true signal position and/or magnitude. This was seen in some cases at 3T and it is possible that subject-specific tuning of RPG registration parameters (λ_2_ and Gaussian smoothing kernel) could have improved the results in these cases. Subject-specific parameter tuning was however not performed in this work, in which we instead showed that improved geometric accuracy can be obtained even though station-wise signal intensities and the amount of susceptibility-induced distortion varies in a WB-DWI examination. The RPG method hence proved robust with regards to registration parameter selection, which can be considered a strength for clinical applicability. A further problem with the RPG method can arise due to fold-over artefacts, which can give rise to singularities in the Jacobian determinant maps and as such inaccuracies in the registration^15^. To avoid fold-over artefacts a large FOV is used in WB-DWI. There is however the potential that very large patients will extend outside the FOV in the phase-encode direction. Depending on the amount of fold-over, the RPG method might not be usable in these cases. All subjects included in this study were however smaller than the FOV, meaning fold-over artefacts were not observed and this effect could not be assessed.

In conclusion, an improved geometric accuracy was obtained when applying the RPG distortion correction on WB-DWI data acquired at 1.5T and 3T. The b0- and b50-based distortion corrections had a very similar performance, which indicates that a b50 EPI acquisition can be used for RPG correction of WB-DWI data and facilitates clinical use.

## Supplementary Information

Supplementary Information.

## Data Availability

The datasets generated during the current study are available from the corresponding author on reasonable request.
