# Supplementary material for: Improved geometric accuracy of whole body diffusion-weighted imaging at 1.5T and 3T using reverse polarity gradients
Source: Sci Rep. 2022 Jul 8;12:11605. doi: 10.1038/s41598-022-15872-6 (PMC9270424; doi:10.1038/s41598-022-15872-6)
Supplement: Supplementary file 1 — Supplementary Information. [file 41598_2022_15872_MOESM1_ESM.pdf]

Improved geometric accuracy of whole body diffusion-weighted imaging at 1.5T and 3T using reverse polarity gradients

T Sjöholm[1]*, J Kullberg[1,2], R Strand[1,3], M Engström[4], H Ahlström[1,2], F Malmberg[1,3]

[1] Department of Surgical Sciences, Uppsala University, Sweden

[2] Antaros Medical AB, Mölndal, Sweden

[3] Department of Information Technology, Uppsala University, Sweden

[4] Applied Science Laboratory, GE Healthcare, Sweden

Supplementary Figure S1. Representative examples of the susceptibility-induced distortion and the effect of distortion correction for sagittal b900 images at 1.5T (top row, a-d) and 3T (bottom row, e-h). Non-corrected (NC) anterior-posterior (AP) (a, e), b0-based distortion corrected (DC-b0) AP (b, f) and b50-based distortion corrected (DC-b50) AP (c, g) images are displayed. Corresponding structural images are also shown (d, h). DWI images are shown in inverted grey-scale with station boundaries marked with a dotted line. Inter-station signal intensity adjustment was performed for the purpose of visualization. Distortion is visible for the spine at station boundaries for NC images. For the distortion corrected data the spine is linked at all station boundaries for both the b0-based and b50-based corrections and its shape better matches the structural image.

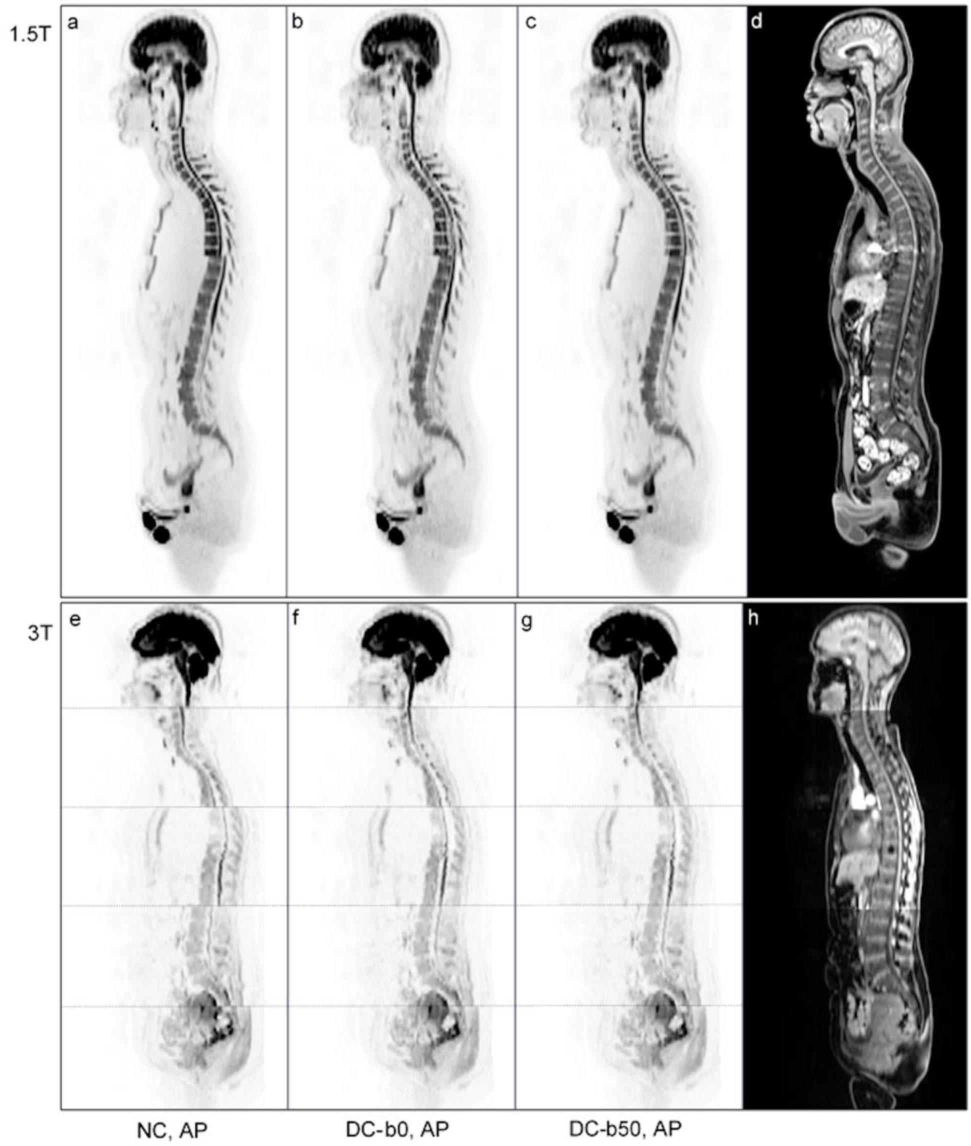

Supplementary Figure S2. Examples of manually drawn ROIs overlaid on structural MR images at 1.5T (top) and 3T (bottom) for one subject with scoliosis (a,c) and one subject with a gallstone (b,d). As indicated by arrows, a mismatch between ROIs defined on non-corrected DWI (NC) and structural MR is evident in particular at 3T. After correction based on b0 (DC-b0) and b50 (DC-b50) data, an improved alignment between ROIs and structural MR is seen.

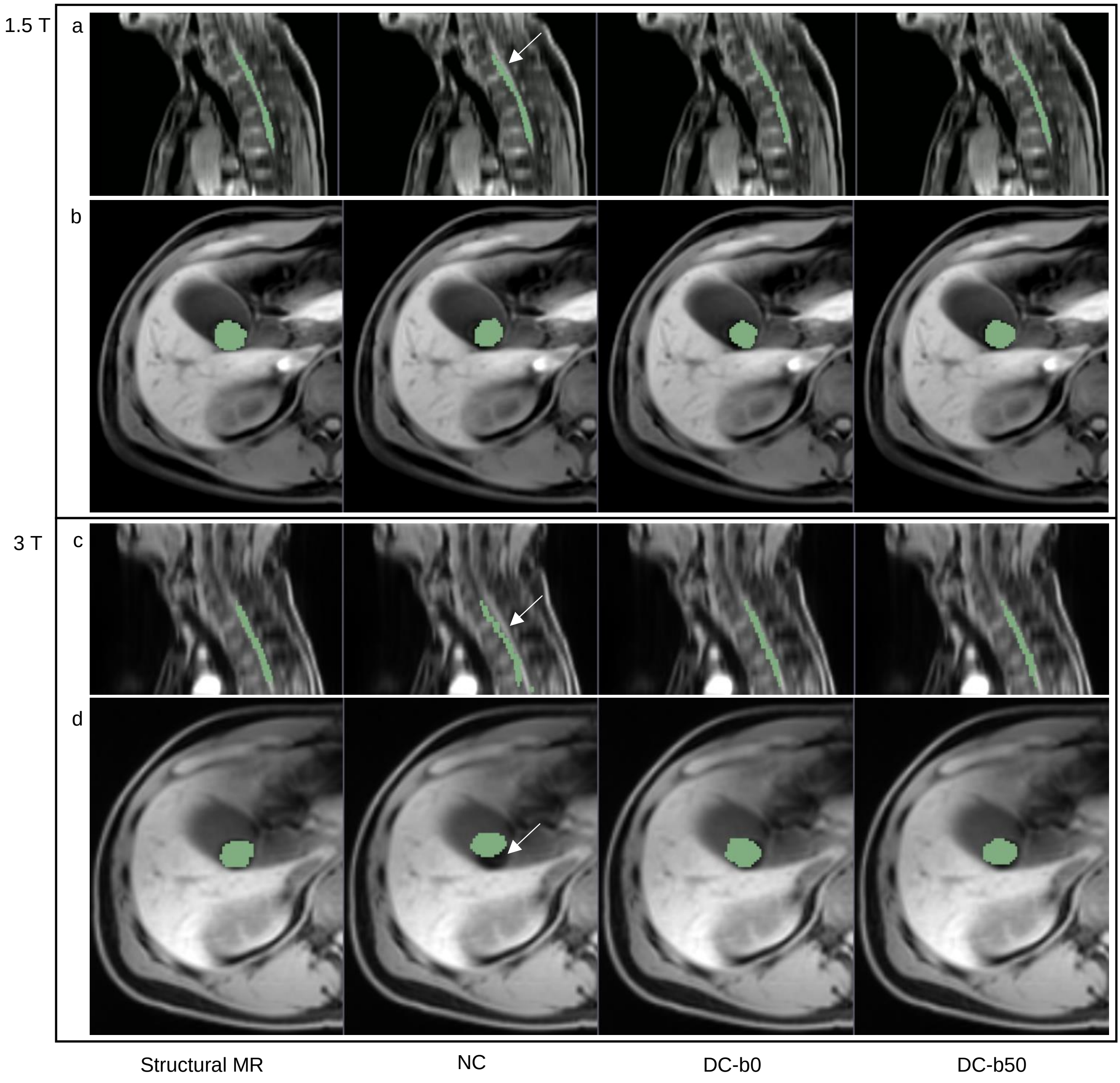

Supplementary Figure 3. Mutual Information (MI) measured station-wise for b900 data. Non-corrected (NC), b0-based distortion corrected (DC-b0) and b50-based distortion corrected (DC-b50) data are shown for 1.5T (left) and 3T (right). P-values obtained when comparing non-corrected and distortion corrected b900 data are shown (Wilcoxon signed-rank test). An asterisk marks tests for which the non-corrected MI>distortion corrected MI.

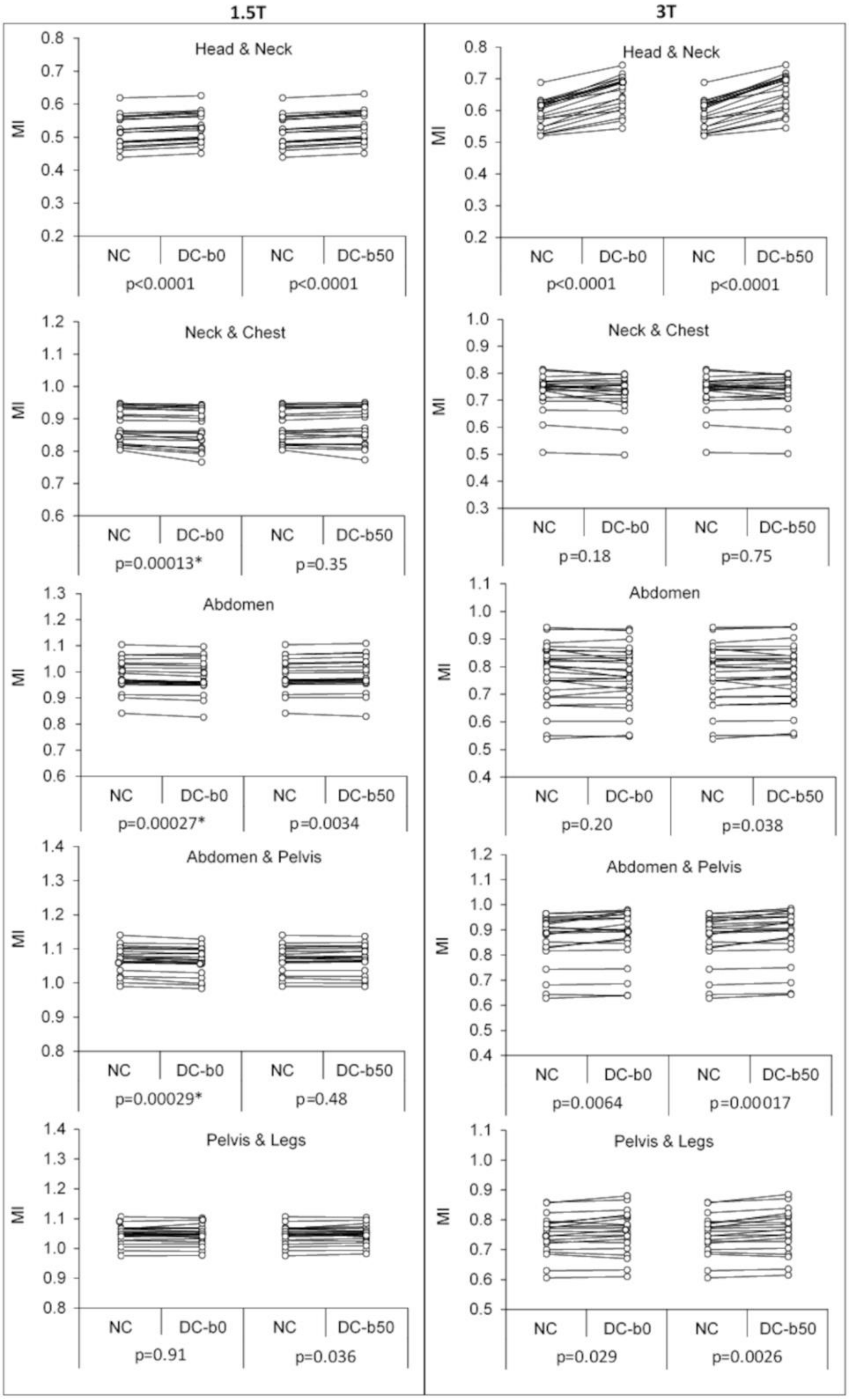

Supplementary Table S1. Comparison between structural MR and DWI by means of the ROI-based metrics Dice Similarity Coefficient (DSC), Euclidean distance of geometric centers (ED) and Average Hausdorff distance (AVD). The difference between results obtained with non-corrected and distortion corrected data is also displayed (Δ). At 3T, the distortion correction gives an overall improved geometrical alignment between ROIs for all metrics. At 1.5T, the overall improvements seen are smaller and when comparing non-corrected and DC-b50 data, no overall improvement was obtained for AVD. At both field strengths, largest improvement was obtained for spinal segmentations, while abdominal segmentations showed small or no improvement (kidney and liver cysts). Subjects scanned at both field strengths are highlighted in bold.

								1.5T							
	DSC					ED					AVD				
	NC	DC-b0	Δ(DC-b0, NC)	DC-b50	Δ(DC-b50, NC)	NC	DC-b0	Δ(DC-b0, NC)	DC-b50	Δ(DC-b50, NC)	NC	DC-b0	Δ(DC-b0, NC)	DC-b50	Δ(DC-b50, NC)
**Scoliosis**	**0.52**	**0.65**	**0.13**	**0.72**	**0.20**	**3.16**	**1.94**	**-1.21**	**2.36**	**-0.80**	**0.81**	**0.41**	**-0.40**	**0.34**	**-0.47**
**Gallstone**	**0.73**	**0.80**	**0.075**	**0.75**	**0.026**	**2.19**	**1.17**	**-1.02**	**1.48**	**-0.71**	**0.42**	**0.30**	**-0.12**	**0.35**	**-0.068**
Splenic cyst	0.63	0.64	0.009	0.58	-0.055	0.97	1.63	0.67	1.88	0.91	0.69	0.60	-0.084	0.84	0.16
**Liver cyst**	**0.64**	**0.58**	**-0.061**	**0.57**	**-0.067**	**1.45**	**0.24**	**-1.21**	**0.59**	**-0.86**	**0.48**	**0.79**	**0.31**	**0.87**	**0.39**
**Kidney cyst**	**0.32**	**0.32**	**-0.0020**	**0.35**	**0.032**	**3.59**	**3.49**	**-0.10**	**3.52**	**-0.075**	**1.53**	**1.36**	**-0.18**	**1.33**	**-0.20**
Kidney cyst	0.38	0.27	-0.11	0.32	-0.065	1.38	2.30	0.92	2.27	0.89	0.78	1.19	0.41	1.09	0.31
Mean (s.d.)	-	-	0.0068 (0.078)	-	0.012 (0.094)	-	-	-0.33 (0.88)	-	-0.11 (0.76)	-	-	-0.0098 (0.28)	-	0.020 (0.30)
								3T							
	DSC					ED					AVD				
	NC	DC-b0	Δ(DC-b0, NC)	DC-b50	Δ(DC-b50, NC)	NC	DC-b0	Δ(DC-b0, NC)	DC-b50	Δ(DC-b50, NC)	NC	DC-b0	Δ(DC-b0, NC)	DC-b50	Δ(DC-b50, NC)
Scoliosis	0.12	0.42	0.30	0.41	0.29	4.64	1.55	-3.09	2.82	-1.82	2.89	1.00	-1.89	1.67	-1.22
**Scoliosis**	**0.43**	**0.68**	**0.25**	**0.67**	**0.24**	**3.83**	**3.04**	**-0.79**	**3.09**	**-0.74**	**0.99**	**0.38**	**-0.61**	**0.38**	**-0.61**
Fat lesion, TX	0.72	0.74	0.025	0.74	0.022	0.83	0.48	-0.35	0.49	-0.34	0.49	0.44	-0.054	0.42	-0.077
**Gallstone**	**0.44**	**0.70**	**0.26**	**0.70**	**0.26**	**4.18**	**2.50**	**-1.69**	**2.45**	**-1.73**	**1.12**	**0.39**	**-0.73**	**0.37**	**-0.76**
Gallstone	0.46	0.47	0.011	0.41	-0.053	1.33	1.05	-0.28	1.03	-0.30	0.89	0.86	-0.028	1.08	0.19
**Liver cyst**	**0.027**	**0.024**	**-0.0033**	**0**	**-0.027**	**7.13**	**8.16**	**1.03**	**8.55**	**1.41**	**4.11**	**5.05**	**0.94**	**5.41**	**1.30**
**Kidney cyst**	**0.28**	**0.28**	**0.0060**	**0.31**	**0.033**	**2.54**	**2.86**	**0.32**	**2.80**	**0.27**	**1.43**	**1.41**	**-0.018**	**1.31**	**-0.12**
Mean (s.d.)	-	-	0.12 (0.13)	-	0.11 (0.14)	-	-	-0.69 (1.26)	-	-0.47 (1.04)	-	-	-0.34 (0.81)	-	-0.18 (0.75)

Supplementary Table S2. Rating and scores given by three radiologists when comparing the image quality of non-corrected and distortion corrected axial b900 images side by side. The number of distortion corrected scans scored as 'equal' or 'worse' compared to the corresponding non-corrected scans are shown for DC-b0 and DC-b50 data. The scores given for scans marked as having worse image quality are also shown. The mean rating and score for the three readers' assessments are indicated. In parentheses, percentages are given.

1.5 T										
	NC vs DC-b0					NC vs DC-b50				
	Equal	Worse	Score 1	Score 2	Score 3	Equal	Worse	Score 1	Score 2	Score 3
Reader 1, n (%)	8 (40)	12 (60)	12 (100)	0 (0)	0 (0)	5 (25)	15 (75)	15 (100)	0 (0)	0 (0)
Reader 2, n (%)	3 (15)	17 (85)	13 (76)	4 (24)	0 (0)	3 (15)	17 (85)	11 (65)	6 (35)	0 (0)
Reader 3, n (%)	11 (55)	9 (45)	4 (44)	3 (33)	2 (22)	6 (30)	14 (70)	11 (79)	3 (21)	0 (0)
Mean, n (%)	7.3 (37)	12.7 (63)	9.7 (76)	2.3 (18)	0.7 (5)	4.7 (23)	15.3 (77)	12.3 (80)	3.0 (20)	0 (0)
3 T										
	NC vs DC-b0					NC vs DC-b50				
	Equal	Worse	Score 1	Score 2	Score 3	Equal	Worse	Score 1	Score 2	Score 3
Reader 1, n (%)	14 (70)	6 (30)	6 (100)	0 (0)	0 (0)	13 (65)	7 (35)	7 (100)	0 (0)	0 (0)
Reader 2, n (%)	9 (45)	11 (55)	9 (82)	2 (18)	0 (0)	5 (25)	15 (75)	11 (73)	4 (27)	0 (0)
Reader 3, n (%)	15 (75)	5 (25)	4 (80)	1 (20)	0 (0)	13 (65)	7 (35)	5 (71)	2 (29)	0 (0)
Mean, n (%)	12.7 (63)	7.3 (37)	6.3 (86)	1.0 (14)	0 (0)	10.3 (52)	9.7 (48)	7.7 (79)	2.0 (21)	0 (0)

NC = non-corrected, DC-b0 = b0-based distortion correction, DC-b50 = b50-based distortion correction

Supplementary Table S3. Visual assessment of b50 and b900 spine misalignment at station boundaries for NC, DC-b0 and DC-b50 data, at 1.5T and 3T, for 14 subjects scanned at both field strengths. The total number of station spine misalignments are displayed per subject. The average misalignment score across all subjects is also given.

	b50						b900					
	NC		DC-b0		DC-b50		NC		DC-b0		DC-b50	
subject	1.5T	3T	1.5T	3T	1.5T	3T	1.5T	3T	1.5T	3T	1.5T	3T
1	0	2	0	1	0	1	0	2	0	1	0	1
2	0	3	0	1	0	0	1	3	0	0	0	0
3	0	2	0	0	0	0	0	2	0	0	0	0
4	1	1	0	0	0	0	0	1	0	0	0	0
5	2	1	0	1	0	1	2	2	0	2	0	2
6	1	1	0	0	0	0	2	2	0	0	0	0
7	1	2	0	0	0	0	1	2	0	0	0	0
8	1	3	0	0	0	0	1	3	0	0	0	1
9	2	2	0	0	0	0	3	2	0	0	0	0
10	0	1	0	0	0	0	0	1	0	0	0	0
11	2	1	0	0	0	0	1	2	0	0	0	0
12	1	3	0	0	0	0	2	3	0	0	0	0
13	1	1	0	0	0	0	1	0	0	0	0	0
14	2	1	0	0	0	0	2	1	0	0	0	0
average misalignment score	1.0	1.7	0.0	0.2	0.0	0.1	1.1	1.9	0.0	0.2	0.0	0.3
